# THE ROLE OF ULTRASONIC CARDIAC OUTPUT MONITOR IN EVALUATING STROKE VOLUME VARIATION TO DETERMINE FLUID RESPONSIVENESS IN PATIENTS WITH SHOCK

**DOI:** 10.1097/SHK.0000000000002584

**Published:** 2025-03-25

**Authors:** Seval Orman, Pervin Hancı, Serdar Efe, Volkan İnal

**Affiliations:** 1 Division of Medical Oncology, Department of Internal Medicine, Turkish Ministry of Health Kartal Doctor Lütfi Kırdar City Hospital, Istanbul, Turkey; 2 Division of Intensive Care Medicine, Department of Pulmonology, Trakya University Faculty of Medicine, Edirne, Turkey; 3 Division of Intensive Care Medicine, Department of Internal Medicine, Uludağ University Faculty of Medicine, Bursa, Turkey; 4 Division of Intensive Care Medicine, Department of Internal Medicine, Trakya University Faculty of Medicine, Edirne, Turkey

**Keywords:** Shock, hemodynamic monitoring, cardiac output, stroke volume, arterial pressure

## Abstract

**Background:** Dynamic assessment of cardiac output (CO) with passive leg raise (PLR), stroke volume variation (SVV), and pulse pressure variation (PPV) offer effective and safe methods to predict fluid responsiveness in patients with shock. The primary aim of this study was to evaluate the reliability of CO and SVV readings with the ultrasonic cardiac output monitor (USCOM) 1A device compared to PPV measurements in determining fluid responsiveness of patients in shock. **Materials and Method:** Intubated and mechanically ventilated patients aged 18–95 with shock admitted to the medical intensive care unit from June 2019 to December 2020 were included in the study. Fluid responsiveness was assessed using PPV from arterial monitoring and CO/SVV using the USCOM 1A device. CO, PPV, and SVV data were recorded before and after PLR. **Results:** Out of 145 shock patients, 92 were included. Before the PLR maneuver, 67 patients had PPV values above 12% and were stated as fluid responsive. The SVV index measured by the USCOM device demonstrated good sensitivity (85%) and specificity (96%) in identifying fluid responsiveness. The agreement with PPV was substantial (Cronbach’s alpha reliability: 0.718 [*P* < 0.001]), and the index was internally consistent (kappa agreement: 0.707 [*P* < 0.001]). The SVV index moderately correlated with PPV (R: 0.588 [*P* = 0.001]). Regarding fluid responsiveness determined by PPV, the AUC value of SVV was 0.797 (0.701–0.894) (p: 0.001). **Conclusion:** SVV measured by the USCOM device is a reliable and practical tool for hemodynamic assessment in clinical practice, particularly when invasive methods are unsuitable.

## INTRODUCTION

Shock is a life-threatening medical condition, caused by various physiological processes that ultimately lead to inadequate cellular oxygenation and perfusion. This critical state poses a challenge for clinicians due to its complex nature and the urgent need for accurate diagnosis and effective treatment. Early recognition and prompt management can significantly improve patient outcomes (1). Regardless of the underlying etiology, the correct management of fluid therapy is vital. Positive fluid balance in critically ill patients increases mortality (2,3), and only half of the patients in shock respond to fluid therapy (4,5). Therefore, the identification of patients who will respond to fluid therapy and the selective application of fluid resuscitation can protect patients from unwanted complications secondary to hypervolemia (6).

Cardiac filling pressures (central venous pressure [CVP] and pulmonary capillary wedge pressure [PCWP]) were traditionally utilized to assess ventricular preload and guide fluid resuscitation. However, these measurements show a poor correlation with blood volume, end-diastolic volumes, and fluid responsiveness (4,7,8). Dynamic parameters, which reflect the variability in stroke volume arising from intrathoracic pressure fluctuations during positive pressure ventilation, have gained attention for evaluating fluid responsiveness. Stroke volume variation (SVV), obtained through pulse contour analysis, and its surrogates, such as pulse pressure variation (PPV) derived from the analysis of the arterial waveform, have been demonstrated to be effective predictors of fluid responsiveness (5,9). These parameters are used in patients who are mechanically ventilated with an adequate tidal volume (≥7–8 mL/kg), without spontaneous breathing, and without arrhythmias. Respiratory variation of more than 12% in these parameters indicates that the patient will respond to fluid therapy, with 79%–88% sensitivity and 84%–89% specificity (10,11). In recent years, efforts have been made to develop less invasive SVV surrogate techniques that do not require invasive arterial catheterization. The Ultrasound Cardiac Output Monitor (USCOM) device is a noninvasive tool for measuring stroke volume and cardiac output (CO) using Doppler ultrasound technology (12). The USCOM device measures the velocity of blood flow exiting the suprasternal region in the aorta and calculates the velocity time integral (VTI), which represents the distance traveled by the blood during a cardiac cycle. Advantages of this method include a reduced risk of infection and other complications associated with invasive procedures, ease of use, the generation of real-time information, and reproducibility (13).

The passive leg-raising maneuver (PLR) is another diagnostic and therapeutic procedure used to assess a patient’s volume status and responsiveness in cases of shock. This reversible test can simulate the effects of a fluid bolus by utilizing gravity to increase venous return and cardiac preload, thereby improving CO in responders. During the PLR maneuver, CO should be measured, and the change in CO before and after the maneuver should be recorded. An increase in CO of 10% or more indicates that the patient’s shock will respond to fluid therapy with 88% sensitivity and 92% specificity (14). Changes in PPV and SVV after the PLR maneuver have also been shown to be important indicators in the assessment of fluid responsiveness (15–17).

This prospective observational study aimed to evaluate the reliability of CO and SVV readings with the USCOM 1A device compared to PPV measurements for determining fluid responsiveness in shock patients. The secondary aim was to investigate the utility of the change in PPV and SVV after PLR in assessing fluid responsiveness.

## MATERIALS AND METHODS

This study was conducted in the 20-bed mixed medical-surgical intensive care unit of a tertiary referral center. Between 1 June 2019 and 30 December 2020, patients aged 18–95 years, who were sedated, intubated, and mechanically ventilated with a tidal volume of at least 7 mL/kg, without spontaneous breathing or arrhythmia, and with clinical signs of hypoperfusion (hypotension, tachycardia, decreased skin turgor and tone, mottled skin, capillary refill time >2 s, confusion, decreased urine output or lactate >2 mmol/L) and diagnosed with shock at ICU admission were included in the study.

The definitions and conventional treatment of septic shock were based on the 2016 European Society of Intensive Care Medicine and Society of Critical Care Medicine guidelines (18). Patients who were under 18 years, pregnant, unable to undergo PLR due to anatomical/physiological stability problems, and with physical/anatomical barriers to USCOM 1A probe insertion were excluded.

### Variables of fluid responsiveness

PPV values of the patients were obtained by routine invasive arterial monitoring procedures with Mindray BeneView T8 monitor systems (Mindray, Shenzhen Mindray Bio-Medical Electronics Co., Ltd., China). PPV was automatically calculated by the monitor’s software as the product of (PPmax − PPmin) / PPmean. After achieving stable values over a defined interval (30 s), values above 12% were deemed fluid-responsive.

CO and SVV measurements were obtained from readings provided by a noninvasive Doppler ultrasonic CO monitor USCOM 1A device (Uscom Ltd., Sydney, Australia). The Doppler probe was placed at the suprasternal notch of the patient. The device automatically calculated CO (SV × HR) and respiratory variation of stroke volume using the formula [(SV_max_ − SV_min_)/SV_mean_]. After obtaining a stable CO and SVV reading, the measurements were recorded; SVV readings above 12% indicated fluid responsiveness. The USCOM measurements were performed by a single experienced operator (VI).

### Passive leg raising maneuver and measurements

For the passive leg raising (PLR) maneuver, the patient is first positioned in a 45-degree, semirecumbent, head-up position. Then, the patient’s upper body is lowered to a horizontal supine position and the legs passively raised 45 degrees upward. The maximum effect occurs after 30–90 s. CO, PPV, and SVV readings were recorded after 90 s of the PLR.

### Data collection

The patient’s gender, age, and shock etiology were noted. CO, PPV, and SVV readings were recorded before and after PLR.

### Statistical analysis

The sample size required for this study was calculated by a power analysis to assess the reliability of the accuracy of the dynamic parameters used to estimate fluid responsiveness measured by the USCOM device. G*Power (ver3.1.9.2 Franz Faul, Universtat Kiel, Germany, 2014) open-source software was used. The effect size was calculated with 50%, two-tailed, error 25%, power 90%, and difference 5. Considering there may be missing data during the collection and processing, it was planned to have at least 40 patients.

Continuous variables were reported as mean ± standard deviation (SD) or median (25th–75th percentile) depending on the normal distribution, while categorical variables were presented as frequencies and proportions (n, %). Demographics and primary admission diagnoses were compared between patients based on fluid responsiveness, which was determined by pulse pressure variation. Fisher’s exact test was used for categorical variables, while The Mann-Whitney *U* test was used for numerical variables. Repeated measures were analyzed using paired samples *t* tests. Pre-PLR PPV and SVV measurements were analyzed by the Bland–Altman method.

Specificity, sensitivity, kappa measure of agreement, and Cronbach alpha reliability were calculated for the CO change with PLR, SVV index to assess their ability to discriminate the volume responsiveness of patients compared to the standard PPV index. Cohen’s kappa coefficient was calculated to assess the classification agreement between two different methods. Kappa values quantitatively assessed the agreement between the methods and the level of agreement was categorized. Cronbach’s alpha coefficient was used to evaluate the internal consistency of the methods.

Correlation and receiver operator curve (ROC) area under the curve (AUC) analysis of CO change with PLR, SVV index, PPV, and SVV change with PLR were also conducted to test their ability to determine volume responsiveness compared to the PPV index. Analyses were two-tailed, confidence intervals (CIs) were measured at 95%, and *P* values <0.05 were considered significant. Determined cutoff values of CO change with PLR and SVV (10% and 12% respectively) for evaluating fluid responsiveness were taken. Youden’s statistics were used to select cutoff values of the delta changes in SVV and PPV with PLR, which gave the best performance by balancing the sensitivity and specifity. The method described by DeLong *et al.* (19) was utilized for the comparison of the ROC curves.

Data management and analyses were performed using the statistical software SPSS (IBM SPSS Statistics ver25, IL, 2017).

### Ethical aspects

The ethical approval for this study was obtained from the Bioethical Board of Trakya University (June 11, 2018—238531). The study was conducted in compliance with the 2008 Declaration of Helsinki. Following the regulatory procedures of the study clinic, patients or legally authorized relatives provided written informed consent for “processing and publishing of patients’ medical records (with names disclosed) for scientific purposes.”

## RESULTS

Out of 145 patients diagnosed with shock during the study period, 92 were included. Fifty-three patients were excluded due to their inability to undergo PLR because of trauma (n = 2) and failure to obtain proper USCOM readings (poor acoustic window) (n = 51). The mean age was 66.9 ± 16.2 (ranging from 19 to 92) years, and the gender distribution was almost equal (45 males and 47 females). Infectious and septic causes are the most common reasons for admission to the ICU. Before the PLR maneuver, 67 patients had PPV values above 12% and were stated as fluid responsive. When patients were grouped as fluid responders and nonfluid responders, no significant difference was observed in terms of age, gender, or admission diagnosis (Table 1).

**Table 1:** Demographics and primary admission diagnoses of patients included in the study according to fluid responsiveness determined by pulse pressure variation

	Fluid responder(n = 69)	Nonfluid responder(n = 23)	*P*
Age*	71 (55–79)	65 (61–77)	0.59†
Gender, male	37 (53.6)	8 (34.8)	0.11‡
Admission diagnoses			0.16‡
*Infection/sepsis*	22 (31.8)	3 (13)	
*Postoperative/trauma*	11 (15.9)	7 (30.4)	
*Cerebrovascular disease*	7 (10.1)	7 (30.4)	
*Cardiopulmonary arrest*	7 (10.1)	3 (13.0)	
*Hematologic emergency*	8 (11.5)	1 (4.3)	
*Oncologic emergency*	6 (8.6)	1 (4.3)	
*Renal failure*	3 (4.3)	1 (4.3)	
*Respiratory failure*	3 (4.3)	0 (0)	
*Gastroenterological emergency*	2 (2.8)	0 (0)	

Data expressed as n (%).

*Median (25–75^th^ percentile).

†Mann-Whitney *U* test

‡Fisher’s exact test.

**Fig. 1:**
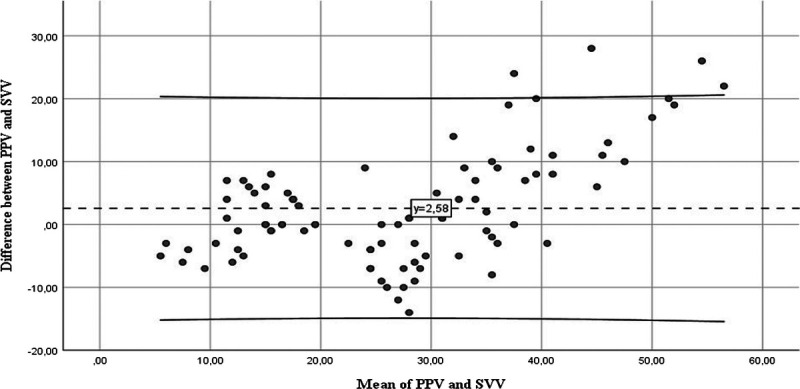
**Bland-Altman analysis of pulse pressure and stroke volume variation for determining fluid responsiveness**. The dashed lines correspond to the mean difference and fit-lines 95% limits of agreement. PPV, pulse pressure variation index; SVV, systolic volume variation index.

The relationship between the differences and averages was analyzed in the Bland-Altman plot to evaluate the agreement between the PPV and SVV measurements before PLR (Fig. 1). The average difference between the two methods was calculated as 2.58%. The limits of agreement (±1.96 SD) are between −14.5 and +19.2, and about 95% of the differences are within these limits. The distribution of the differences was observed as randomly distributed.

Fifty-seven patients had SVV values above 12%. After the PLR, more than a 10% increase in CO was observed in 66 patients. CO, SVV, and PPV values before and after PLR are presented in Table 2. The mean CO was increased (delta CO: 0.56 ± 0.38 [t: 14.1, *P* < 0.001]), while there were reductions in both PPV (delta PPV: 4.7 ± 3.0 [t: 6.8, p: 0.001]), and SVV (delta PPV: 5.8 ± 4.4 [t: 10.2, p: 0.001]) values with the PLR maneuver.

**Table 2:** Pre- and post-PLR PPV, CO, and SVV are presented as means ± SD and differences of two consequence measurements

	Mean ± SD	Difference	t	*P*
PrePLR-PPV	18.9 ± 10.1	4.7 ± 3.0	6.8	0.001*
PostPLR-PPV	14.2 ± 8.4			
PrePLR-CO	2.63 ± 1.47	0.56 ± 0.38	14.1	<0.001*
PostPLR-CO	3.20 ± 1.53			
PrePLR-SVV	16.4 ± 6.9	5.8 ± 4.4	10.2	0.001*
PostPLR-SVV	10.5 ± 3.8			

*Paired-samples *t* test.

CO, cardiac output; PLR, passive leg raising maneuver; PPV, pulse pressure variation index; SVV, stroke volume variation index.

PPV was used as the standard test for comparisons. Crosstabs analyses tested the discriminative ability of CO change with PLR and SVV, as contingency tables were presented in Table 3. We then proceeded with specificity, sensitivity, kappa measure of agreement, and Cronbach’s alpha calculations. CO change with PLR measured by the USCOM device had 77% sensitivity and 40% specificity (positive predictive value: 77%, negative predictive value: 40%), showed unacceptable Cronbach’s alpha reliability levels (0.295 [*P* = 0.12]), and showed nonsignificant and slight kappa agreement values (0.176 [*P* = 0.09]). SVV index had 85% sensitivity and 96% specificity (positive predictive value: 98%, negative predictive value: 68%), had acceptable Cronbach’s alpha reliability levels (0.718 [*P* < 0.001]), and showed substantial kappa agreement values (0.707 [*P* < 0.001]) for discriminating pre-PLR volume responsive status.

**Table 3:** Presentation of specificity, sensitivity, agreement, and reliability of CO change with PLR and SVV index to discriminate volume responsiveness statutes of patients compared to standard PPV index

	Specificity	Sensitivity	Kappa measure of agreement	*P*	Cronbach’s alpha	*P*
CO change with PLR	40	77	0.176	0.09*	0.295	0.12†
SVV	96	83	0.707	<0.001*	0.718	0.000†

*Z test.

†F test.

CO, cardiac output, PPV, pulse pressure variation index; SVV, stroke volume variation index.

**Fig. 2:**
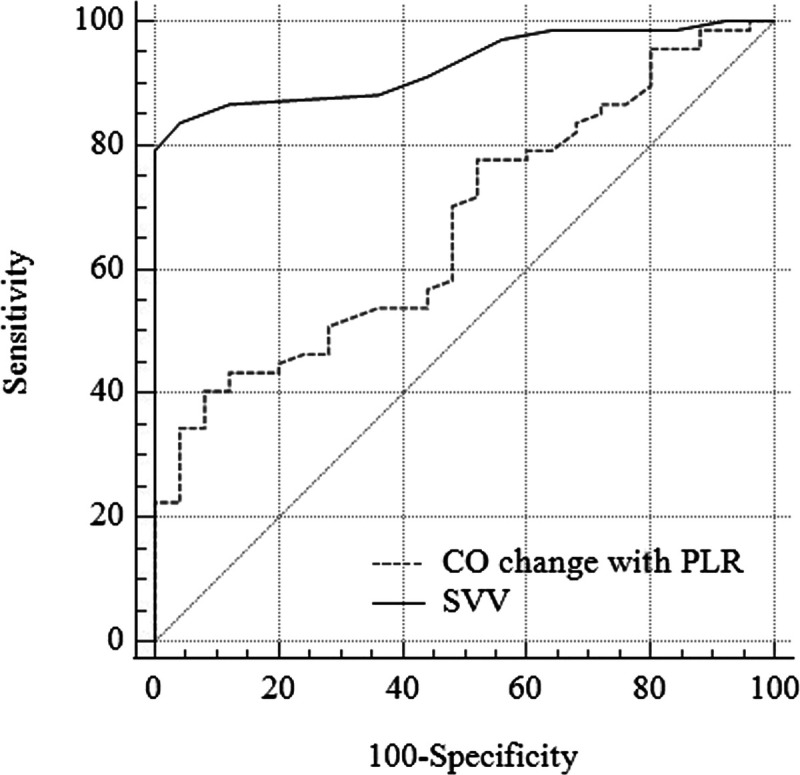
**ROC curve for CO change with PLR and SVV predicting volume responsiveness determined by PPV index.** CO, cardiac output; PLR, passive leg raise; PPV, pulse pressure variation index; ROC, receiver operator characteristics; SVV, systolic volume variation index.

**Fig. 3:**
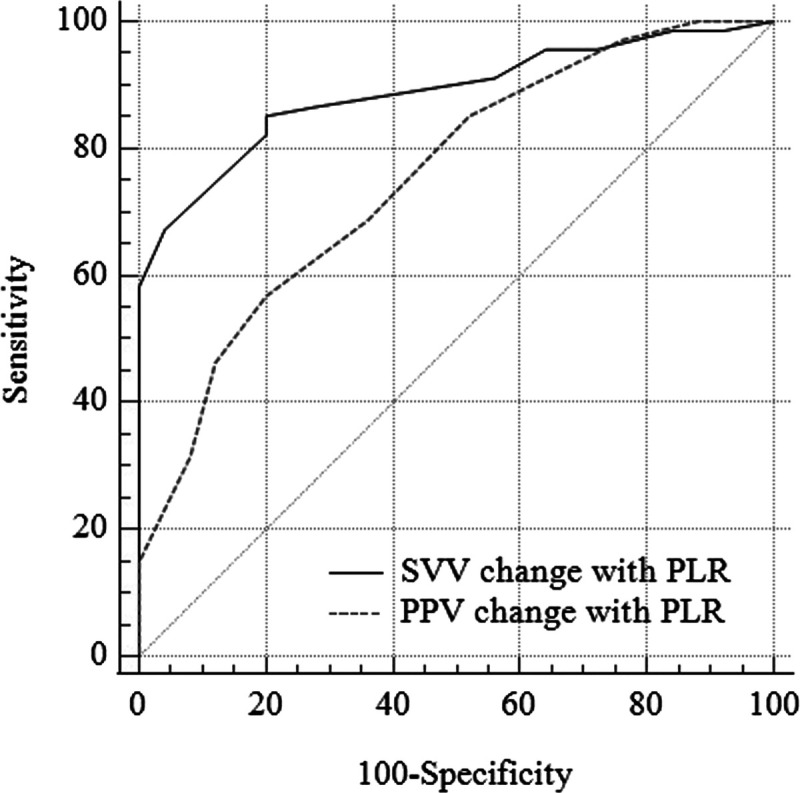
**ROC curve for SVV change with PLR predicting volume responsiveness determined by PPV index.** CO, cardiac output; PLR, passive leg raise; PPV, pulse pressure variation index; ROC, receiver operator characteristics; SVV, systolic volume variation index.

Correlation and ROC AUC analyses results of the SVV index; CO, SVV, and PPV change with PLR measurements for standard PPV readings are presented in Table 4 and Figures 2 and 3. Compared to CO change with the PLR maneuver (0.671 [0.565–0.765]), SVV had a higher AUC value (0.797 [0.701–0.894]) for determining fluid responsiveness (*P* < 0.01). SVV also had a better correlation with PPV. Regarding fluid responsiveness determined by PPV, the AUC values for delta changes in SVV and PPV with PLR were 0.887 (95% CI: 0.805–0.944) (*P* < 0.001) and 0.755 (95% CI: 0.655–0.839) (*P* < 0.001), respectively. The optimal cutoff values and corresponding sensitivity and specificity for delta changes in SVV and PPV with PLR were 3 (sensitivity: 85%, specificity 80%) and 4 (sensitivity: 56%, specificity: 80%), respectively.

**Table 4:** Correlation and ROC AUC analysis of CO change with PLR and SVV to test the ability of volume responsiveness determined by the PPV index

	Correlation		ROC			Comparison of ROC curves
	r	*P*	AUC	95% CI	*P*	*P*
CO	0.232	0.02	0.671	0.565–0.765	0.004*	<0.001†
SVV	0.588	0.001	0.797	0.701–0.894	0.001*	
SVV change with PLR	0.594	<0.001	0.887	0.805–0.944	<0.001*	0.03†
PPV change with PLR	0.663	<0.001	0.755	0.655–0.839	<0.001*	

*ROC curve analysis.

†DeLong test.

PPV, pulse pressure variation index; ROC AUC, receiver operator characteristics area under curve; SVV, systolic volume variation index.

## DISCUSSION

This study aimed to evaluate the diagnostic performance of CO change with PLR and SVV indices, as measured by the USCOM device, for identifying fluid responsiveness in comparison with the standard PPV index. The SVV index measured by the USCOM device demonstrated good sensitivity and excellent specificity in identifying fluid responsiveness. The agreement with PPV was substantial, and the index was internally consistent. The SVV index had a significant moderate correlation with PPV. The ROC AUC suggested that the SVV measured by USCOM was a good predictor for volume responsiveness, as determined by standard PPV. This study also revealed that a 3% decrease in SVV during the PLR maneuver accurately predicted fluid responsiveness.

Critically ill patients require continuous and close monitoring of hemodynamics to guide therapies. Although invasive hemodynamic monitoring methods are considered the gold standard, noninvasive monitoring techniques offer advantages in terms of accuracy and precision, while reducing the risk of complications. Noninvasive devices have been successfully implemented, particularly in certain patient groups. However, the use of noninvasive CO monitoring devices that can provide accurate and reliable measurements in the intensive care unit is still limited, which presents a significant challenge in clinical practice.

The USCOM is an increasingly popular noninvasive device used for monitoring CO. The USCOM offers several advantages over invasive methods, including convenience, low risk, and enhanced patient comfort. (13,20) Many studies have been carried out to evaluate the accuracy and range of applications of the USCOM device. Tan *et al.* performed 40 paired measurements in 22 patients with stable hemodynamics and compared USCOM measurements with CO values obtained by the thermodilution technique in a pulmonary artery catheter (PAC). They reported that the USCOM monitor underestimated CO (20). In another study conducted in patients with unstable hemodynamics, a good correlation was reported between CO measurements with PAC and USCOM (r = 0.80 / 0.64), but underestimation of CO was up to 30%, especially with higher flows (21). A study conducted in septic shock patients found high agreement (r = 0.89) in CO measured by USCOM and transpulmonary thermodilution, but with a 29% margin of error (22). In 2018, a meta-analysis study involving 844 patients across 22 different studies examined USCOM data compared to data obtained from other invasive reference methods (23). The analysis revealed a high level of heterogeneity among the studies (*I*^2^ = 95%, *P* < 0.001). Additionally, the percentage error was found to be 38%, which exceeds the clinically acceptable threshold of 30% (24). Therefore, it has been reported that CO values obtained with the USCOM device cannot be considered a reliable alternative to standard reference methods, such as bolus thermodilution CO monitoring. Possible probe relocation, estimation errors in the valve area, and user mistakes may contribute to the high percentage of error. Consequently, monitoring CO in intensive care using an insufficiently sensitive USCOM device is controversial. Although the USCOM device may show reliability in some populations, in our study, its sensitivity in evaluating CO change during the PLR test was found to be moderate (77%), and the specificity was very low (40%). Both Cronbach’s alpha and kappa coefficients were very low and not statistically significant in terms of reliability or consistency. These results suggest that the device’s performance in this specific test is unreliable and does not provide consistent results.

However, the accurate prediction of changes in hemodynamics is more important than the measured value itself in assessing fluid responsiveness in critically ill patients. Detecting a respiratory change in stroke volume with a single measurement may therefore be as valuable as knowing the change in CO for predicting fluid responsiveness (25,26). This can be measured directly, or estimated through arterial pressure waveform analysis, with PPV as an alternative. SVV effectively predicts preload dependency, but its accuracy is influenced by tidal volume (24). Studies have compared SVV measurements using different systems, such as FloTrac/Vigileo and PiCCOplus, finding similar performance in predicting fluid responsiveness (27). SVV is useful not only in patients with normal cardiac function, but also in those with reduced left ventricular function after cardiac surgery (28). In patients with reduced cardiac function, SVV correlates significantly with changes in stroke volume index caused by volume loading and can predict volume responsiveness comparably to other hemodynamic parameters, such as pulmonary artery occlusion pressure and CVP.

The USCOM device can also calculate SVV and has demonstrated high sensitivity and specificity for predicting fluid responsiveness in children after congenital heart surgery (29). When compared to esophageal Doppler monitoring during major abdominal surgery, USCOM showed good concordance and accurately detected changes in SVV ≥10% (30). These findings suggest that USCOM could be a valuable tool for noninvasive hemodynamic assessment in various clinical settings, although its limitations should be considered when interpreting results. In our study, we determined fluid responsiveness using PPV as the reference method and tried to determine the predictive power of SVV measured by USCOM.

The high sensitivity and specificity of the SVV index (89%) suggest that it is highly effective in accurately identifying fluid responsiveness in patients with shock. This makes it particularly valuable in clinical settings, where identifying fluid-responsive patients is critical for the optimization of hemodynamic management and the avoidance of unnecessary interventions. The balance of sensitivity and specificity suggests that SVV is well suited for initial screening, particularly in hemodynamically unstable patients. The slight agreement between SVV and PPV reflects their comparable performance in evaluating volume responsiveness, albeit with some variability. Given that PPV is a well-established standard, the observed agreement supports the validity of the SVV index measured by USCOM as an alternative method. This finding is particularly important in clinical scenarios where invasive methods of deriving PPV are impractical or contraindicated. Furthermore, the acceptable internal consistency of the SVV index suggests its reliability as a measurement tool. The moderate correlation between SVV and PPV underscores a significant association between these indices. The ROC AUC value of 0.797 further confirms the utility of SVV as a predictor of volume responsiveness, with the AUC exceeding the threshold for good discriminative ability. This suggests that the SVV index measured by USCOM can reliably differentiate between responders and nonresponders, a critical requirement for bedside hemodynamic monitoring. The performance of SVV, as demonstrated in this study, aligns with previous research emphasizing its utility in predicting fluid responsiveness.

The decrease in PPV and SVV following the PLR maneuver may indicate fluid responsiveness. Mallat *et al.* assessed the effectiveness of cardiac index (CCI), PPV, and SVV changes in predicting fluid responsiveness after mini fluid loading in 49 critically ill patients who were on mechanical ventilation with low tidal volume (15). Each patient received 100 mL of colloid within 1 min, followed by 400 mL of fluid over 14 min. The aim was to classify patients as responders if they exhibited an increase of 15% or more in CI after the complete (500 mL) fluid loading. CCI, PPV, and SVV values were compared before and after fluid loading. The increase in cardiac index after 100 mL of fluid (with a cutoff point of 5.2%) was moderately effective in predicting fluid responsiveness, achieving an AUC of 0.78. However, there was a significant gray zone (67%), indicating uncertainty of outcome for many patients. In contrast, changes in PPV (−2%) and SVV (−2%) demonstrated higher accuracy in predicting fluid responsiveness, with AUCs of 0.91 and 0.92, respectively. The gray zones for these parameters were smaller (less than 12%), and they exhibited better sensitivity (86%) and specificity (85%–89%). Ma *et al.* also showed that changes in SVV from the PLR maneuver could predict fluid responsiveness in patients undergoing protective mechanical ventilation after cardiac surgery (17). In that study, a 4% decrease in SVV with PLR effectively predicted fluid responsiveness, achieving a high accuracy (AUC: 0.90).

Our study observed significant decreases in PPV (delta PPV: 4.7 ± 3.0, *P* = 0.001) and SVV (delta SVV: 5.8 ± 4.4, *P* = 0.001) following the PLR maneuver. We found that the change in the SVV index after PLR had very good discriminative ability for predicting fluid responsiveness according to PPV; a decrease of 3 or more was both highly sensitive (85%) and highly specific (80%). These findings support the notion that PLR is a reliable method for evaluating hemodynamic response through SVV parameters, consistent with existing literature. Notably, the significant changes in SVV values highlight the potential of PLR for accurately predicting fluid responsiveness. Our study reinforces the current literature and suggests that these parameters should be integrated into clinical decision-making processes for patients under protective mechanical ventilation.

While the findings of this study are encouraging, several limitations must be acknowledged. Firstly, we did not use standard reference methods, such as pulmonary or transpulmonary thermodilution, to assess fluid responsiveness. Instead, we relied on PPV as the reference for fluid responsiveness. Although PPV is well-validated, its accuracy can be affected by factors such as reduced lung or chest wall compliance and interactions between the right and left ventricles. Secondly, all measurements using the USCOM device were performed by a single, experienced operator. This may limit the reproducibility of the results with other operators. Third, while the majority of patients in our study were in septic shock, the sample size and heterogeneity of the patient group may complicate the assessment of the accuracy specific to the etiology of shock.

In conclusion, the SVV index measured by the USCOM device demonstrated strong sensitivity, specificity, and good predictive ability in identifying volume responsiveness, with moderate correlation with the PPV index. These findings suggest that SVV measured by the USCOM device is a reliable and practical tool for hemodynamic assessment in clinical practice, particularly when invasive methods are unsuitable. However, its limitations necessitate cautious interpretation and, ideally, complementary use with other indices to guide fluid management decisions.

Authors’ contributions: Vİ and SE searched the literature. Vİ, SE, and SO designed the study. SE and SO enrolled the patients in the study, performed the measurements, and assessed data entry. PH and Vİ performed the statistical analysis. PH and Vİ prepared the manuscript and drafted the article. All authors interpreted the data. PH takes full responsibility for the work as a whole, including access to data and the decision to submit and publish the manuscript. All authors approved this manuscript in its final form.
